# Incidental Detection of Respiratory Viruses in Lung Transplant Donor‐Recipient Pairs: Exploratory Associations With Clinical Outcomes in a Post Hoc Analysis

**DOI:** 10.1111/tid.70275

**Published:** 2026-07-10

**Authors:** Andrea Lombardi, Letizia Corinna Morlacchi, Giulia Renisi, Lorenzo Rosso, Jacopo Fumagalli, Patrizia Bono, Ilaria Righi, Valeria Rossetti, Arianna Liparoti, Cecilia Azzarà, Davide Mangioni, Laura Alagna, Anna Celotti, Claudia Alteri, Chiara Abbruzzese, Annapaola Callegaro, Francesco Blasi, Giacomo Grasselli, Mario Nosotti, Alessandra Bandera

**Affiliations:** ^1^ SC Malattie Infettive Fondazione IRCCS Ca’ Granda Ospedale Maggiore Policlinico Milano Italy; ^2^ Dipartimento Di Fisiopatologia Medico‐Chirurgica e dei Trapianti Università degli Studi di Milano Milano Italy; ^3^ SC Pneumologia e Fibrosi Cistica Fondazione IRCCS Ca’ Granda Ospedale Maggiore Policlinico Milano Italy; ^4^ SC Chirurgia Toracica e Trapianti di Polmone Fondazione IRCCS Ca’ Granda Ospedale Maggiore Policlinico Milano Italy; ^5^ SC Anestesia e Terapia Intensiva Adulti Fondazione IRCCS Ca’ Granda Ospedale Maggiore Policlinico Milano Italy; ^6^ SC Microbiologia e Virologia Fondazione IRCCS Ca’ Granda Ospedale Maggiore Policlinico Milano Italy

**Keywords:** Community‐acquired respiratory viruses, Lung transplantation, Syndromic molecular panel

## Abstract

**Background:**

Infections caused by community‐acquired respiratory viruses (CARV) are very common among lung transplant recipients and have been linked to acute rejection and chronic lung allograft dysfunction (CLAD). However, the clinical relevance of CARV detection in donor‐recipient pairs at the time of lung transplantation remains unclear.

**Methods:**

Post hoc analysis of the Pneumoarray study, evaluating the clinical significance of incidentally detected CARV identified by syndromic molecular panels (PN*plus*) in BAL samples collected 72 h after transplantation from donors and recipients.

**Results:**

Among 53 donor‐recipient pairs, CARV were identified in 7 (13.2%) and 11 (20.8%) BAL samples from donors and recipients, respectively. Unadjusted analyses showed a link between CARV PN*plus* positivity in donor samples and longer hospitalization (Δ+30.3 days, 95% CI 7.1–53.5; *p* = 0.011). Any CARV PN*plus* positivity during the transplant episode also indicated a potential link with longer hospital stays (Δ+19.2 days, 95% CI 1.4–37.0; *p* = 0.035). Donor CARV PN*plus* positivity showed a signal suggesting a potential association with higher odds of CLAD within 12 months (OR 8.40, 95% CI 0.84–83.89; *p* = 0.030). CARV PN*plus* positivity in the recipient for rhinovirus indicated a potential link with baseline lung allograft dysfunction (*p* = 0.016), while no other relationships were observed between clinical outcomes and being PN*plus* positive for a viral pathogen.

**Conclusion:**

CARV genome detection in donor BAL samples was numerically associated with longer posttransplant hospitalization, and a potential signal between rhinovirus detection in recipient BAL and baseline lung allograft dysfunction was observed. However, these findings should be regarded as exploratory and hypothesis‐generating, and they do not support the routine baseline screening for CARV in lung transplant donor‐recipient pairs.

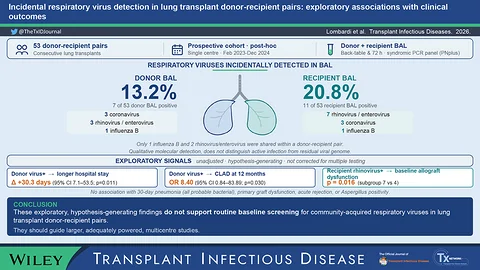

## Graphical abstract

In 53 lung transplant donor‐recipient pairs, community‐acquired respiratory viruses were incidentally detected by syndromic PCR in 13.2% of donor and 20.8% of recipient bronchoalveolar lavages, with exploratory, unadjusted signals towards longer hospitalization and chronic lung allograft dysfunction. These hypothesis‐generating findings do not support routine baseline viral screening at the time of lung transplantation.

**Abbreviations**
AAR: acute antibody rejection
ACR: acute cellular rejection
BAL: bronchoalveolar lavage
BLAD: baseline lung allograft dysfunction
CARV: community‐acquired respiratory viruses
CF: cystic fibrosis
CLAD: chronic lung allograft dysfunction
COPD: chronic obstructive pulmonary disease
DCD: donation after circulatory death
FEV1: forced expiratory volume
ICU: intensive care unit
ILD: interstitial lung disease
LOS: length of stay
LUTR: LuTx recipients
LuTx: lung transplantation
PGD: primary graft dysfunction
PN*plus*
: BioFire FilmArray Pneumonia Panel Plus
SOC: standard of care

## 1 Introduction

Infections due to community‐acquired respiratory viruses (CARV) are extremely common among lung transplant recipients (LUTR) and represent up to 80% of total viral infections in the first year after transplantation [1]. They can evolve into bronchiolitis and pneumonia following lung transplantation (LuTx) and may serve as risk factors for secondary bacterial and fungal infections [2]. Interestingly, CARV infections have been associated with both acute rejection and chronic lung allograft dysfunction (CLAD) [2, 3, 4, 5].

However, the impact of pretransplant CARV infections on posttransplant outcomes in LuTx recipients remains a matter of debate. Recent experiences have not detected any association between the presence of CARV, assessed by nucleic acid amplification test on routine bronchoscopy specimens performed on Day 1 posttransplant, and the development of Grade 3 primary graft dysfunction (PGD) within the first 72 h [6]. On the other hand, current guidelines recommend screening symptomatic LuTx candidates for CARV infections using polymerase chain reaction (PCR) testing and obtaining symptom resolution before proceeding with LuTx [7].

In this study, we aimed to evaluate the clinical relevance of CARV detection in bronchoalveolar lavage (BAL) samples obtained from lung transplant donor–recipient pairs in the peri‐transplant period. We performed a post hoc analysis of a prospective cohort study assessing the performance of a syndromic molecular panel among LuTx candidate‐recipient pairs.

## 2 Methods

The Pneumoarray study is a prospective investigation comparing the syndromic molecular panel BioFire Filmarray Pneumonia Panel Plus (PN*plus*) with traditional culture‐based methods for the microbiological diagnosis of BAL samples from LuTx donors and recipients. The study included all the consecutive LuTx performed at the LuTx Centre of the Fondazione IRCCS Ca’ Granda Ospedale Maggiore Policlinico of Milan, Italy, in the period from February 1, 2023 to December 31, 2024.

This post hoc analysis of the Pneumoarray study was designed to assess the clinical relevance of incidentally detected viral pathogens included in the PN*plus* assay, in donor and recipient BAL samples collected 72 h after LuTx, in accordance with the internal protocol.

The PN*plus* is a multiplex PCR assay detecting 15 common bacterial pathogens, three atypical bacteria, eight respiratory viruses, and seven genetic resistance determinants. Resistance genes detected are reported only when a potential carrier bacterium is present. Common bacteria are reported semi‐quantitatively (10^4^–10^7^ genomic copies/mL), while atypical bacteria, viruses, and resistance determinants are reported qualitatively (detected/not detected). The list of targets identified by PN*plus* is provided in Table S1. Standard microbiological diagnostics and bronchoscopy were performed as previously described. Donor BAL was obtained during back‐table inspection of the procured lungs by bronchoscope through the main bronchus before implantation, whereas recipient BAL was obtained by surveillance bronchoscopy within 72 h of LuTx in the recipient's allograft, sampling the same anatomical region; both samples were processed identically. Routine virological testing of BAL was not part of standard of care: CARV PCR was performed only when clinically indicated, and SARS‐CoV‐2, which is not included in the PN*plus* panel, was tested separately only when clinically indicated. PN*plus* reports respiratory viruses qualitatively, without Ct values or copy numbers [8]. For each patient, demographic and clinical data were extracted from electronic records.

The complete list of definitions used for PGD, antibody‐mediated rejection (AMR), acute cellular rejection (ACR), baseline lung allograft dysfunction (BLAD), and CLAD is provided in the Supporting Information.

Continuous variables were summarized as mean ± standard deviation (SD) and median with interquartile range (IQR), as appropriate. Between‐group comparisons were performed using the Mann–Whitney *U* test for continuous variables. Categorical variables were reported as counts and percentages and compared using Pearson's chi‐square test or Fisher's exact test, as appropriate. Unadjusted logistic regression models were used to estimate odds ratios (ORs) with 95% confidence intervals (CIs) for binary outcomes, while linear regression models were applied to assess associations between viral isolation and length‐of‐stay outcomes. Due to the limited number of outcome events, multivariable adjustment was not performed to avoid model overfitting. All tests were two‐tailed, and *p*‐values < 0.05 were considered statistically significant. Analyses were performed using STATA version 19 (StataCorp, College Station, TX, USA).

Written informed consent was obtained from all participants; the study was approved by the local ethical committee (665_2022bis), registered on ClinicalTrials.gov (NCT05960383), and conducted in accordance with the Helsinki Declaration.

## 3 Results

The study enrolled 60 LuTx candidates. Of these, seven were excluded because PN*plus* was not performed on both the donor's and the recipient's samples due to logistical constraints. Overall, 53 donor‐recipient pairs are included in the current analysis (Figure S1).

Males constituted the majority (62.3%) of this cohort, with a mean age at LuTx of 52.7 years; interstitial lung disease and cystic fibrosis/bronchiectasis were the most common indications for transplantation, being the underlying lung disease in 15 and 12 patients, respectively. Most cases involved double LuTx (88.7%), with 10 instances of donor donation after circulatory death. LuTxs were distributed across all seasons (Winter 9, Spring 12, Summer 20, Autumn 12), and CARV detection was not concentrated in winter (Table 1).

**TABLE 1:** Clinical characteristics of lung transplant recipients at the time of transplantation.

	Overall (*n* = 53)	LuTx viral PN*plus* negative (*n* = 38)	LuTx viral PN*plus* positive (*n* = 15)	*p*
Gender				
Male, *n* (%)	33 (62.3%)	23 (60.5%)	10 (66.7%)	0.761
Female, *n* (%)	20 (37.7%)	15 (39.5%)	5 (33.3%)	
Age	52.7 ± 12.4	54.8 ± 11.5	47.4 ± 13.3	0.071
BMI, Kg/m^2^ (SD)	24.2 ± 4.2	25.2 ± 4.1	21.8 ± 3.7	**0.007**
Fraction of inspired O_2_ at rest pretransplant, (SD)	15.9 ± 14.3	15.8 ± 14.6	16.2 ± 13.9	0.927
FEV1, L (SD)	1.77 ± 3.60	1.3 ± 0.6	3 ± 6.7	0.343
Donor donation after circulatory death, *n* (%)	10 (18.9%)	6 (15.8%)	4 (26.6%)	0.442
DCD2, *n* (%)	5 (9.4%)	3 (7.9%)	2 (13.3%)	0.614
DCD3, *n* (%)	5 (9.4%)	3 (7.9%)	2 (13.3%)	
Type of transplantation				
Single lung, *n* (%)	6 (11.3%)	4 (10.5%)	2 (13.3%)	1
Double lung, *n* (%)	47 (88.7%)	34 (89.5%)	13 (86.7%)	
Transplant indication				
Cystic fibrosis and bronchiectasis, *n* (%)	12 (22.6%)	9 (23.7%)	3 (20.0%)	0.142
ILD, *n* (%)	15 (28.3%)	13 (34.2%)	2 (13.3%)	
Connective tissue disease, *n* (%)	6 (11.3%)	4 (10.5%)	2 (13.3%)	
COPD, *n* (%)	8 (15.1%)	6 (15.8%)	2 (13.3%)	
Lymphangioleiomyomatosis and sarcoidosis, *n* (%)	2 (3.8%)	0	2 (13.3%)	
Other, *n* (%)	16 (30.2%)	9 (23.7%)	7 (46.7%)	
Comorbidities				
Diabetes, *n* (%)	9 (17%)	9 (23.7%)	0	**0.035**
Obesity, *n* (%)	3 (5.7%)	3 (7.9%)	0	0.540
Chronic liver disease, *n* (%)	3 (5.7%)	3 (7.9%)	0	0.540
Chronic heart disease, *n* (%)	7 (13.2%)	5 (13.2%)	2 (13.3%)	1
Charlson Comorbidity Index				
0, *n* (%)	1 (1.9%)	1 (2.6%)	0	0.103
1, *n* (%)	10 (18.9%)	4 (10.5%)	6 (40.0%)	
2, *n* (%)	13 (24.5%)	9 (23.7%)	4 (26.7%)	
3, *n* (%)	20 (37.7%)	18 (47.4%)	2 (13.3%)	
4, *n* (%)	7 (13.2%)	5 (13.2%)	2 (13.3%)	
5, *n* (%)	2 (3.8%)	1 (2.6%)	1 (6.7%)	

Abbreviations: BMI, body mass index; COPD, chronic obstructive pulmonary disease; FEV1, forced expiratory volume; ILD, interstitial lung disease.

In the donor's BAL sample, PN*plus* was positive in 36 (67.9%) cases, and CARV were identified in 7 (13.2%) patients. Overall, three coronaviruses, three human rhinoviruses/enteroviruses, and one influenza B virus were identified. The recipient with influenza received preemptive treatment with oseltamivir 75 mg twice daily for 10 days (Table 2A, Figure S2).

**TABLE 2:** Viral isolation on donor's (A) and recipient's (B) bronchoalveolar lavage sample, according to the BioFire Filmarray Pneumonia Panel Plus (PN*plus*).

*N*	53
**(A)**	
PN*plus* positive overall, *n* (%)	36 (67.9%)
PN*plus* positive > 1 overall target, *n* (%)	18 (34%)
Time to results PN*plus*, minutes (SD)	221.8 ± 387.8
PN*plus* positive for viruses, *n* (%)	7 (13.2%)
PN*plus* positive > 1 viral target, *n* (%)	0
Species identified	
Coronavirus, *n* (%)	3 (42.9%)
Human rhinovirus/enterovirus, *n* (%)	3 (42.9%)
Influenza B, *n* (%)	1 (14.3%)
**(B)**	
PN*plus* positive overall, *n* (%)	32 (60.4%)
PN*plus* positive > 1 overall target, *n* (%)	15 (28.3%)
Time to results PN*plus*, minutes (SD)	157.7 ± 229.2
PN*plus* positive for viruses, *n* (%)	11 (20.8%)
PN*plus* positive > 1 viral target, *n* (%)	0
Species identified	
Human rhinovirus/enterovirus, *n* (%)	7 (63.3%)
Coronavirus, *n* (%)	3 (33.3%)
Influenza B, *n* (%)	1 (9.1%)

In contrast, in the recipient's BAL sample, PN*plus* was positive in 32 cases (60.4%), and CARV were identified in 11 patients (20.8%). Overall, seven human rhinoviruses/enteroviruses, three coronaviruses, and one influenza B virus were identified. Notably, only the influenza B virus and two human rhinoviruses/enteroviruses were isolated in both donor and recipient samples (Table 2B).

PN*plus* positivity in donor samples showed a signal suggesting a possible association with longer recipient hospitalization (mean 67.3 vs. 37.0 days; unadjusted Δ +30.3 days, 95% CI 7.1–53.5; *p* = 0.011). Any PN*plus* positivity during the transplant episode (LuTx PN*plus*) was also linked to longer recipient hospital stays (Δ +19.2 days; *p* = 0.035). Donor PN*plus* positivity showed a trend suggesting a possible link with higher odds of CLAD within 12 months (OR 8.40, 95% CI 0.84–83.89; *p* = 0.030). Notably, the incidence of pneumonia in the 30 days following LuTx was similar between groups, and all identified episodes were recognized as having a probable bacterial etiology (Table 3).

**TABLE 3:** Association between clinical outcomes and BioFire Filmarray Pneumonia Panel Plus (PN*plus*) positive for viral pathogen. (Values are *n* (%) unless otherwise specified. Lengths of stay reported as mean ± SD; median [IQR]. Odds ratios [OR] are unadjusted with 95% CI) Given the small subgroup sizes, ORs and *p*‐values are reported for transparency only and should be interpreted as exploratory; lengths of stay are summarized by median [IQR].

Outcome	Donor viral PN*plus* negative (*n* = 46)	Donor viral PN*plus* positive (*n* = 7)	OR (95%CI); *p*	Recipient viral PN*plus* negative (*n* = 42)	Recipient viral PN*plus* positive (*n* = 11)	OR (95% CI); *p*	LuTx viral PN*plus* negative (*n* = 38)	LuTx viral PN*plus* positive (*n* = 15)	OR (95% CI); *p*
**Early outcomes**									
PGD	16 (34.8%)	3 (42.9%)	1.41 (0.27–7.20); *p* = 0.681	13 (31.0%)	6 (54.5%)	2.68 (0.66–10.81); *p* = 0.150	12 (31.6%)	7 (46.7%)	1.90 (0.54–6.60); *p* = 0.307
ICU length of stay, days	7.9 ± 11.1; 4 [3–6]	16.4 ± 18.9; 12 [2–28]	Δ +8.5 days; *p* = 0.096	8 ± 11.9; 3 [3–7]	12.9 ± 14.7; 4 [4–28]	Δ +4.9 days; *p* = 0.258	7 ± 9.7; 3 [3–6]	14.2 ± 17; 4 [3–28]	Δ +7.2 days; *p* = 0.060
Hospital length of stay, days	37.0 ± 20.6; 30 [22–48]	67.3 ± 60.1; 48 [19–144]	Δ +30.3 days (7.1–53.5); ***p* = 0.011**	38.4 ± 26.7; 30 [21–48]	51.0 ± 40; 37 [19–71]	Δ +12.6 days; *p* = 0.219	35.5 ± 18.5; 30 [22–44]	54.7 ± 46; 37 [19–71]	Δ +19.2 days (1.4–37.0); ***p* = 0.035**
Pneumonia ≤ 30 days	11 (24.4%)	3 (42.9%)	2.32 (0.43–12.40); *p* = 0.312	12 (29.3%)	2 (18.2%)	0.54 (0.10–2.94); *p* = 0.466	10 (27.0%)	4 (26.7%)	0.98 (0.25–3.86); *p* = 0.979
Infection ≤ 30 days	15 (32.6%)	3 (42.9%)	1.55 (0.30–7.98); *p* = 0.597	14 (33.3%)	4 (36.4%)	1.14 (0.28–4.63); *p* = 0.852	12 (31.6%)	6 (40.0%)	1.44 (0.41–5.07); *p* = 0.564
Antibiotics ongoing at Day 7	32 (69.6%)	7 (100%)	*p* = 0.092	31 (73.8%)	8 (72.7%)	0.95 (0.21–4.28); *p* = 0.943	27 (71.1%)	12 (80.0%)	1.63 (0.38–7.06); *p* = 0.510
Antibiotics ongoing at Day 14	30 (65.2%)	6 (85.7%)	3.20 (0.34–30.31); *p* = 0.284	27 (64.3%)	9 (81.8%)	2.50 (0.46–13.59); *p* = 0.272	24 (63.2%)	12 (80.0%)	2.33 (0.54–10.04); *p* = 0.241
In‐hospital death	2 (4.3%)	0 (0%)	*p* = 0.578	1 (2.4%)	1 (9.1%)	0.24 (0.01–4.49); *p* = 0.303	1 (2.6%)	1 (6.7%)	0.38 (0.02–6.74); *p* = 0.492
Death ≤ 30 days	2 (4.3%)	0 (0%)	*p* = 0.578	1 (2.4%)	1 (9.1%)	4.10 (0.22–75.56); *p* = 0.303	1 (2.6%)	1 (6.7%)	2.64 (0.15–47.06); *p* = 0.492
**12‐month outcomes**									
BLAD	16 (36.4%)	2 (28.6%)	0.70 (0.12–4.11); *p* = 0.692	12 (29.3%)	6 (60.0%)	3.63 (0.81–16.19); *p* = 0.071	11 (29.7%)	7 (50.0%)	2.36 (0.64–8.66); *p* = 0.181
CLAD	2 (4.5%)	2 (28.6%)	8.40 (0.84–83.89); ***p* = 0.030**	2 (4.9%)	2 (20.0%)	4.88 (0.55–43.05); *p* = 0.114	2 (5.4%)	2 (14.3%)	2.92 (0.35–24.07); *p* = 0.297
AAR	4 (9.1%)	1 (14.3%)	1.67 (0.15–18.03); *p* = 0.671	4 (9.8%)	1 (10.0%)	1.03 (0.10–10.59); *p* = 0.982	4 (10.8%)	1 (7.1%)	0.63 (0.06–6.39); *p* = 0.697
ACR	9 (20.5%)	2 (28.6%)	1.56 (0.25–9.58); *p* = 0.631	8 (19.5%)	3 (30.0%)	1.77 (0.36–8.59); *p* = 0.474	8 (21.6%)	3 (21.4%)	0.99 (0.22–4.49); *p* = 0.988

Abbreviations: AAR, acute antibody rejection; ACR, acute cellular rejection; BLAD, baseline lung allograft dysfunction; CLAD, chronic lung allograft dysfunction; ICU, intensive care unit; LuTx, lung transplantation; PGD, primary graft dysfunction.

Interestingly, PN*plus* positivity in the recipient for rhinovirus indicated a potential link with BLAD (*p* = 0.016), while no other connections between clinical outcomes and PN*plus* positivity for a viral pathogen were discovered, whether stratified by rhinovirus versus other respiratory viruses identified in donor BAL, recipient BAL, or overall BAL samples (Tables S2–S4).

The median pretransplant length of stay (interval between hospital admission and LuTx) was 1 day in all CARV groups, reflecting the institutional protocol of admitting candidates on the day before the procedure. Although the mean pretransplant LOS was numerically longer in donor CARV‐positive recipients (6.3 vs. 2.3 days), this difference was not statistically significant (*p* = 0.276). After adjustment for pretransplant LOS in a linear regression model, the association between donor CARV positivity and posttransplant total hospital LOS remained substantial (adjusted Δ +34.1 days, 95% CI 12.9–55.4; *p* = 0.002). A sensitivity analysis excluding the single influenza B donor‐recipient pair confirmed that the association between donor CARV positivity and hospital LOS persisted and was slightly stronger (Δ +41.0 days, 95% CI 19.0–63.0; *p* < 0.001).

Posttransplant *Aspergillus* spp. positivity (galactomannan and/or PCR on respiratory samples) was observed in 9/52 (17%) recipients. Numerically, *Aspergillus* positivity was more frequent in donor CARV‐positive recipients (3/8, 37.5%) than in donor CARV‐negative recipients (6/46, 13.0%), without reaching statistical significance (*p* = 0.118), and no association was observed when stratifying by recipient CARV or any‐BAL CARV positivity. Invasive mold infection within 30 days was recorded in 3/19 evaluable patients, with no signal of differential incidence across CARV strata.

## 4 Discussion

In this prospective cohort of LuTx candidate‐recipient pairs undergoing syndromic molecular panel testing, CARV were identified in 13% of BAL samples from donors and 20% from recipients. Donor CARV positivity showed a signal suggesting a possible association with longer hospitalization (+30 days; *p* = 0.011) and showed a potential association with CLAD (OR 8.40; *p* = 0.030). Recipient viral positivity showed trends toward associations with higher BLAD risk and longer LOS. Overall, a viral positivity in the donor‐recipient pair indicated a potential association with longer hospitalization (*p* = 0.035) and showed a borderline link with ICU stay (*p* = 0.060). Finally, PN*plus* positivity in the recipient for rhinovirus showed a possible association with BLAD (*p* = 0.016), whereas no other associations were observed between clinical outcomes and being PN*plus*‐positive for a viral pathogen.

The association between CLAD development and CARV infection is supported by several studies [4, 9, 10, 11], with different viruses linked to this complication; however, none have investigated the significance of CARV infection prospectively in the perioperative setting. It is worth noting that the majority of studies associated the development of CLAD with relevant CARV infections (pneumonia or lower respiratory tract disease), rather than with the mere identification of the microorganism, as in our case. Moreover, Fisher et al. suggested a putative positive correlation between the proximity of CLAD development and LuTx, which aligns well with our preliminary data [12].

This study has several important limitations. First, the small sample size resulted in limited statistical power and wide CIs, reducing the precision of effect estimates. Second, the absence of multivariable adjustment precludes accounting for potential confounding factors, such as donor characteristics, recipient severity, or perioperative complications. Third, viral detection was based on molecular assays, which do not distinguish between active infection and the presence of non‐viable viral genomes. Fourth, SARS‐CoV‐2 is not included in the PN*plus* panel and was tested separately only when clinically indicated. Fifth, the marked right‐skewness of length‐of‐stay distributions makes parametric estimates of effect size unstable and supports a strictly nonparametric interpretation of these comparisons. The diagnostic equivalence of PN*plus* to conventional multiplex respiratory virus NAT assays has been previously documented [13]. Finally, we did not test for the presence of the CARV genome in recipients immediately before LuTx, and thus we cannot precisely determine which CARV were already present in recipients.

Overall, our exploratory data suggest a possible association between CARV genome detection in donor BAL samples and a numerically longer posttransplant hospital stay, although the small sample size, the post hoc design, and the multiplicity of comparisons preclude any causal inference. Importantly, when total hospital LOS was adjusted for pretransplant LOS, the donor CARV‐positive effect persisted, arguing against a confounding role of nosocomial CARV acquisition during a longer pretransplant inpatient stay; a sensitivity analysis excluding the single influenza B donor‐recipient pair likewise confirmed the stability of the signal. *Aspergillus* positivity was numerically more frequent in donor CARV‐positive recipients, but the difference was not statistically significant, and no association was detected for invasive mold infection within 30 days.

The reasons behind these results are difficult to hypothesize considering the sample size and the design of this study. We can speculate that the presence of a virus, potentially in active replication, in the donor's lung parenchyma can create an inflammatory milieu. In this condition, the administration of immunosuppressive drugs can support a persistent infection status, which, in turn, could sustain the release of pathogen‐associated molecular patterns and inflammatory cytokines. This is followed by an intricate interplay between innate and adaptive immune responses, with infiltration of innate and adaptive immune cells into the allograft, ultimately leading to fibrosis and organ dysfunction [14]. It must be acknowledged that recent evidence shows that rhinovirus, the most frequently identified pathogen in our cohort, can replicate in the lower respiratory tract, contrary to the traditional belief that it is limited to the upper airways, underscoring its potential involvement in the inflammatory process [15].

In our exploratory subgroup analysis, recipient rhinovirus detection showed a possible signal towards BLAD. A biologically plausible, although speculative, interpretation is that rhinovirus replication in the lower airway epithelium of an immunosuppressed recipient could sustain persistent epithelial signaling, impaired mucociliary clearance, and low‐grade innate immune activation, thereby facilitating early allograft injury even in the absence of overt clinical symptoms. Nevertheless, given the very small subgroup (7 vs. 4) and the multiplicity of tested outcomes, this association must be regarded as hypothesis‐generating and is in itself insufficient to support any change in current clinical management.

These exploratory findings do not support the systematic baseline screening for CARV in donors or recipients at the time of LuTx, nor any change in current clinical practice. They should be regarded as hypothesis‐generating and used solely to inform the design of larger, adequately powered, multicenter studies.

Overall, our preliminary results should be considered exploratory and hypothesis‐generating; their primary value is to motivate larger, adequately powered, multicenter prospective studies that systematically assess the presence and the actual infectivity of CARV in LuTx donor‐recipient pairs at the time of organ procurement, together with relevant clinical and immunological outcomes, before any change in clinical management can be considered.

## Author Contributions

A.L., L.M., G.R., and A.B. conceived the study. G.R., C.A. and A.Li. collected the clinical data. A.L. performed the statistical analyses. A.L. wrote the first draft of the manuscript. All the authors reviewed the final version of the manuscript.

## Funding

This study was partially supported by IRCCS Ricerca Corrente 2026 funds.

## Conflicts of Interest

The authors declare no conflicts of interest.

## Supporting information




**Supplementary Figure 1**: Study flowchart. **Supplementary Figure 2**: Sankey's diagram illustrating the results of BioFire Filmarray Pneumonia Panel Plus (PN*plus*) for community‐acquired respiratory viruses in donor and recipient BAL samples. **Supplementary Table 1**: List of targets detected by BioFire Filmarray Pneumonia Panel Plus (PN*plus*) according to the producer. **Supplementary Table 2**: Association between clinical outcomes and the BioFire Filmarray Pneumonia Panel Plus (PN*plus*) positive for a community‐acquired respiratory virus, stratified by rhinovirus vs other respiratory viruses identification on donor BAL. (Values are n (%) unless otherwise specified. **Supplementary Table 3**: Association between clinical outcomes and the BioFire Filmarray Pneumonia Panel Plus (PN*plus*) positive for a community‐acquired respiratory virus, stratified by rhinovirus vs other respiratory viruses identification on recipient BAL. (Values are n (%) unless otherwise specified. **Supplementary Table 4**: Association between clinical outcomes and the BioFire Filmarray Pneumonia Panel Plus (PN*plus*) positive for a community‐acquired respiratory virus, stratified by rhinovirus vs other respiratory viruses identification on any BAL sample. (Values are n (%) unless otherwise specified.

**Supporting File**: Visual Abstract.

## References

[1] C. Van Delden , S. Stampf , H. H. Hirsch , et al., “Burden and Timeline of Infectious Diseases in the First Year After Solid Organ Transplantation in the Swiss Transplant Cohort Study,” Clinical Infectious Diseases 71 (2020): E159–E169, 10.1093/cid/ciz1113. PMC7583409 31915816

[2] M. Peghin , H. H. Hirsch , Ó. Len , et al., “Epidemiology and Immediate Indirect Effects of Respiratory Viruses in Lung Transplant Recipients: A 5‐Year Prospective Study,” American Journal of Transplantation 17 (2017): 1304–1312, 10.1111/ajt.14042. PMC7159570 27615811

[3] D. Kumar , S. Husain , M. H. Chen , et al., “A Prospective Molecular Surveillance Study Evaluating the Clinical Impact of Community‐Acquired Respiratory Viruses in Lung Transplant Recipients,” Transplantation 89 (2010): 1028–1033, 10.1097/TP.0b013e3181d05a71. 20075787

[4] M. Peghin , I. Los‐Arcos , H. H. Hirsch , et al., “Community‐Acquired Respiratory Viruses Are a Risk Factor for Chronic Lung Allograft Dysfunction,” Clinical Infectious Diseases 69 (2019): 1192–1197, 10.1093/cid/ciy1047. PMC7797743 30561555

[5] A. Weinberg , D. M. Lyu , S. Li , J. Marquesen , and M. R. Zamora , “Incidence and Morbidity of Human Metapneumovirus and Other Community‐Acquired Respiratory Viruses in Lung Transplant Recipients,” Transplant Infectious Disease 12 (2010): 330–335, 10.1111/j.1399-3062.2010.00509.x. PMC7169861 20456714

[6] L. van den Bosch , K. Kaur , R. Varughese , et al., “Respiratory Viruses at Time of Lung Transplant and the Relationship to Primary Graft Dysfunction,” Transplant Infectious Disease 27 (2025): e70078, 10.1111/tid.70078. PMC12519916 40717602

[7] F. Riunga , C. Cervera , and D. Kabbani , “Pretransplant Screening for Infections in Lung and Heart Transplant Candidates,” JHLT Open 11 (2026): 100409, 10.1016/j.jhlto.2025.100409. PMC12677184 41356173

[8] A. Lombardi , G. Renisi , L. Rosso , et al., “Performance of the BioFire FilmArray Pneumonia Panel Plus Compared to Standard Microbiology in Lung Transplant Donor and Recipient Samples: A Prospective Cohort Study,” Transplant Infectious Disease 28 (2026): e70186, 10.1111/tid.70186. PMC13262558 41719008

[9] H. Testaert , M. Bouet , F. Valour , et al., “Incidence, Management and Outcome of Respiratory Syncytial Virus Infection in Adult Lung Transplant Recipients: A 9‐Year Retrospective Multicentre Study,” Clinical Microbiology and Infection 27 (2021): 897–903, 10.1016/j.cmi.2020.07.050. 32827713

[10] P. R. Allyn , E. L. Duffy , R. M. Humphries , et al., “Viral Pneumonia Accelerates Graft Death and CLAD in Lung Transplant Recipients,” Journal of Heart and Lung Transplantation 34 (2015): S305, 10.1016/j.healun.2015.01.858.

[11] A. De Zwart , A. Riezebos‐Brilman , G. Lunter , et al., “Respiratory Syncytial Virus, Human Metapneumovirus, and Parainfluenza Virus Infections in Lung Transplant Recipients: A Systematic Review of Outcomes and Treatment Strategies,” Clinical Infectious Diseases 74 (2022): 2252–2260, 10.1093/cid/ciab969. PMC9258934 35022697

[12] C. E. Fisher , C. M. Preiksaitis , E. D. Lease , et al., “Symptomatic Respiratory Virus Infection and Chronic Lung Allograft Dysfunction,” Clinical Infectious Diseases 62 (2016): 313–319, 10.1093/cid/civ871. PMC4706632 26565010

[13] D. M. Webber , M. A. Wallace , C. A. D. Burnham , and N. W. Anderson , “Evaluation of the Biofire Filmarray Pneumonia Panel for Detection of Viral and Bacterial Pathogens in Lower Respiratory Tract Specimens in the Setting of a Tertiary Care Academic Medical Center,” Journal of Clinical Microbiology 58 (2020): e00343–e00420, 10.1128/JCM.00343-20. PMC7315030 32321782

[14] S. Bos , L. Milross , A. J. Filby , R. Vos , and A. J. Fisher , “Immune Processes in the Pathogenesis of Chronic Lung Allograft Dysfunction: Identifying the Missing Pieces of the Puzzle,” European Respiratory Review 31 (2022): 220060, 10.1183/16000617.0060-2022. PMC9724884 35896274

[15] R. Zhang , X. Shang , C. Wang , et al., “Rhinovirus‐Associated Lower Respiratory Tract Infection in Hospitalized Adult Patients: A Retrospective Cohort Study,” Journal of Infectious Diseases 233 (2025): 868–876, 10.1093/infdis/jiaf651. 41457011

