# Supplementary material for: Incidental Detection of Respiratory Viruses in Lung Transplant Donor‐Recipient Pairs: Exploratory Associations With Clinical Outcomes in a Post Hoc Analysis
Source: Transpl Infect Dis. 2026 Jul 10;28(5):e70275. doi: 10.1111/tid.70275 (PMC13638784; doi:10.1111/tid.70275)
Supplement: Supplementary file 1 [file TID-28-e70275-s001.docx]

**Incidental detection of respiratory viruses in lung transplant donor-recipient pairs: exploratory associations with clinical outcomes in a post-hoc analysis**

**Figures legends**

**Supplementary Figure 1.** Study flowchart.

**Supplementary Figure 2.** Sankey’s diagram illustrating the results of BioFire Filmarray Pneumonia Panel Plus (PN*plus*) for community-acquired respiratory viruses in donor and recipient BAL samples.

**Supplementary Table 1.** List of targets detected by BioFire Filmarray Pneumonia Panel Plus (PN*plus*) according to the producer.

| **Gram-positive** | *Staphylococcus aureus* |
| --- | --- |
|  | *Streptococcus pneumoniae* |
|  | *Streptococcus pyogenes* |
|  | *Streptococcus agalactiae* |
|  |  |
| **Gram-negative** | *Escherichia coli* |
|  | *Enterobacter cloacae* |
|  | *Klebsiella aerogenes* |
|  | *Proteus* spp. |
|  | *Klebsiella pneumoniae* group |
|  | *Klebsiella oxytoca* |
|  | *Serratia marcescens* |
|  | *Moraxella catarrhalis* |
|  | *Pseudomonas aeruginosa* |
|  | *Acinetobacter calcoaceticus-baumannii* complex |
|  | *Legionella pneumophila* |
|  | *Haemophilus influenzae* |
|  |  |
| **Viruses** | Adenovirus |
|  | Coronavirus |
|  | Metapneumovirus |
|  | Rhinovirus/Enterovirus |
|  | Influenza A |
|  | Influenza B |
|  | Parainfluenza virus |
|  | RSV |
|  | MERS-CoV |
|  |  |
| **Atypical pathogens** | *Chlamydophila pneumoniae* |
|  | *Mycoplasma pneumoniae* |
|  |  |
| **Antibiotic resistance genes** | Carbapenemases: KPC, NDM, OXA-48 like, VIM, IMP |
|  | ESBL: CTX-M |
|  | Methicillin resistance: mecA/mecC and MREJ |

RSV, respiratory syncytial virus; MERS-CoV, Middle East Respiratory Syndrome coronavirus; KPC, Klebsiella pneumoniae carbapenemase; NDM, New Delhi metallo-beta-lactamase; VIM, Verona integron-encoded metallo-beta-lactamase; IMP, imipenemase; MREJ, mec element right extremity junction.

**Supplementary Table 2.** Association between clinical outcomes and the BioFire Filmarray Pneumonia Panel Plus (PN*plus*) positive for a community-acquired respiratory virus, stratified by rhinovirus vs other respiratory viruses identification on donor BAL. (Values are n (%) unless otherwise specified. Lengths of stay reported as mean ± SD; median [IQR]. Odds ratios (OR) are unadjusted with 95%CI.) Given the small subgroup sizes, ORs and p-values are reported for transparency only and should be interpreted as exploratory; lengths of stay are summarised by median [IQR].

| **Outcome** | **rhinovirus (n=3)** | **Other viruses* (n=4)** | **OR (95%CI); p** |
| --- | --- | --- | --- |
| **PGD** | 2 (66.7%) | 1 (25.0%) | 6.00 (0.12–302.38); p=0.307 |
| **BLAD** | 1 (33.3%) | 1 (25.0%) | 1.50 (0.04–53.72); p=0.823 |
| **CLAD** | 1 (33.3%) | 1 (25.0%) | 1.50 (0.04–53.72); p=0.823 |
| **AAR** | 0 (0%) | 1 (25.0%) | p=0.387 |
| **ACR** | 1 (33.3%) | 1 (25.0%) | 1.50 (0.04–53.72); p=0.823 |
| **Infection ≤30 days** | 1 (33.3%) | 2 (50.0%) | 0.50 (0.02–14.90); p=0.683 |
| **Pneumonia ≤30 days** | 1 (33.3%) | 2 (50.0%) | 0.50 (0.02–14.90); p=0.683 |
| **Hospital LOS (days)** | 72.5 ± 32.7 | 60.3 ± 37.8 | Δ −12.2 days; p=0.817 |
| **ICU LOS (days)** | 20.3 ± 10.0 | 11.3 ± 11.6 | Δ −8.9 days; p=0.586 |

*Other respiratory viruses include coronavirus and influenza B.

PGD, primary graft dysfunction; BLAD, baseline lung allograft dysfunction; CLAD, chronic lung allograft dysfunction; AAR, acute antibody rejection; ACR, acute cellular rejection; ICU, intensive care unit; LuTx, lung transplantation.

**Supplementary Table 3.** Association between clinical outcomes and the BioFire Filmarray Pneumonia Panel Plus (PN*plus*) positive for a community-acquired respiratory virus, stratified by rhinovirus vs other respiratory viruses identification on recipient BAL. (Values are n (%) unless otherwise specified. Lengths of stay reported as mean ± SD; median [IQR]. Odds ratios (OR) are unadjusted with 95%CI.) Given the small subgroup sizes, ORs and p-values are reported for transparency only and should be interpreted as exploratory; lengths of stay are summarised by median [IQR].

| **Outcome** | **rhinovirus (n=7)** | **Other viruses* (n=4)** | **OR (95%CI); p** |
| --- | --- | --- | --- |
| **PGD** | 5 (71.4%) | 1 (25.0%) | 7.50 (0.28–199.25); p=0.156 |
| **BLAD** | 6 (85.7%) | 0 (0%) | **p=0.016** |
| **CLAD** | 1 (14.3%) | 1 (25.0%) | 0.33 (0.01–10.57); p=0.513 |
| **AAR** | 0 (0%) | 1 (25.0%) | p=0.127 |
| **ACR** | 2 (28.6%) | 1 (25.0%) | 0.80 (0.04–17.20); p=0.886 |
| **Infection ≤30 days** | 2 (28.6%) | 2 (50.0%) | 0.40 (0.03–6.22); p=0.498 |
| **Pneumonia ≤30 days** | 1 (14.3%) | 1 (25.0%) | 0.50 (0.02–13.28); p=0.673 |
| **Hospital LOS (days)** | 62.7 ± 14.5 | 30.5 ± 19.2 | Δ +32.2 days; p=0.215 |
| **ICU LOS (days)** | 17.0 ± 5.4 | 5.8 ± 7.1 | Δ +11.3 days; p=0.240 |

*Other respiratory viruses include coronavirus and influenza B.

PGD, primary graft dysfunction; BLAD, baseline lung allograft dysfunction; CLAD, chronic lung allograft dysfunction; AAR, acute antibody rejection; ACR, acute cellular rejection; ICU, intensive care unit; LuTx, lung transplantation.

**Supplementary Table 4.** Association between clinical outcomes and the BioFire Filmarray Pneumonia Panel Plus (PN*plus*) positive for a community-acquired respiratory virus, stratified by rhinovirus vs other respiratory viruses identification on any BAL sample. (Values are n (%) unless otherwise specified. Lengths of stay reported as mean ± SD; median [IQR]. Odds ratios (OR) are unadjusted with 95%CI.) Given the small subgroup sizes, ORs and p-values are reported for transparency only and should be interpreted as exploratory; lengths of stay are summarised by median [IQR].

| **Outcome** | **rhinovirus (n=8)** | **Other viruses* (n=6)** | **OR (95%CI); p** |
| --- | --- | --- | --- |
| **PGD** | 5 (62.5%) | 1 (16.7%) | 8.33 (0.41–169.56); p=0.098 |
| **BLAD** | 6 (75.0%) | 1 (16.7%) | 12.00 (0.42–341.10); p=0.063 |
| **CLAD** | 1 (12.5%) | 1 (16.7%) | 0.57 (0.02–13.63); p=0.726 |
| **AAR** | 0 (0%) | 1 (16.7%) | p=0.206 |
| **ACR** | 2 (25.0%) | 1 (16.7%) | 1.33 (0.08–22.57); p=0.842 |
| **Infection ≤30 days** | 2 (25.0%) | 3 (50.0%) | 0.33 (0.03–3.79); p=0.352 |
| **Pneumonia ≤30 days** | 1 (12.5%) | 2 (33.3%) | 0.29 (0.02–5.15); p=0.365 |
| **Hospital LOS (days)** | 57.1 ± 13.0 | 34.2 ± 15.0 | Δ +23.0 days; p=0.269 |
| **ICU LOS (days)** | 15.1 ± 4.7 | 6.3 ± 5.4 | Δ +8.8 days; p=0.242 |

*Other respiratory viruses include coronavirus and influenza B.

PGD, primary graft dysfunction; BLAD, baseline lung allograft dysfunction; CLAD, chronic lung allograft dysfunction; AAR, acute antibody rejection; ACR, acute cellular rejection; ICU, intensive care unit; LuTx, lung transplantation.

**Definitions employed in the study.**

Primary graft dysfunction (PGD) refers to the syndrome of acute lung injury early after lung transplantation and is clinically characterised by diffuse alveolar infiltrates on chest X-ray imaging, with the degree of associated hypoxemia determining its severity. Severe PGD (PGD3), in particular, is defined as a PaO2:FiO2 ratio of < 200 at 48 or 72 h (with a window of +/- 6 h) after reperfusion.[1,2]

Antibody mediated rejection (AMR) diagnosis currently relies on a combination of circulating DSA, evidence of complement activation, histological pattern, graft dysfunction and exclusion of other diagnoses, such as acute cellular rejection (ACR) or infection; the last ISHLT consensus paper includes three levels of diagnostic certainty (definite, probable and possible AMR) as well as definitions for clinical and sub-clinical AMR.[3,4]

Acute cellular lung rejection (ACR) is diagnosed on the histological observation of lymphocytic infiltrates in a perivascular (acute perivascular inflammation) or peribronchiolar (lymphocytic bronchiolitis) distribution. Standardised criteria for the diagnosis and severity grading of acute perivascular inflammation and lymphocytic bronchiolitis, based on the degree of lymphocytic infiltration, have been established by the International Society for Heart and Lung Transplantation (ISHLT).[5,6] Of note, the term ACR represents a spectrum of disease that encompasses a vastly heterogeneous group of histological, physiological, and clinical entities with potentially unique biological underpinnings.[2] For this study, we considered as “clinically relevant episodes of ACR” all the following: ISHLT Grade ≥A2; or ISHLT Grade ≥A1 with either a decline in FEV1 of at least 10% from recent baseline, or other clinical evidence of graft injury, such as new breathlessness, ground glass opacification on HRCT or new oxygen requirement.

Baseline lung allograft dysfunction (BLAD) is defined as the failure to achieve normal peak lung transplant function. BLAD is a common condition, detected in 42% of LUTRs. BLAD is determined by longitudinal spirometry after bilateral LuTx and is defined as failure to ever achieve≥80%-predicted forced expiratory volume in 1 second (FEV1) and forced vital capacity (FVC) 1. BLAD is associated with an increased risk of death related to physiologic vulnerability initiated by poor baseline lung function.[7] Risk factors for BLAD include native interstitial lung disease, high-grade primary graft dysfunction (PGD3), and heavy donor smoking.[7,8]

Finally, chronic lung allograft dysfunction (CLAD) is a clinical syndrome of progressive loss of lung allograft function from established baseline after lung transplantation, not explained by other conditions, and, as per ISHLT consensus paper, is defined as a substantial and persistent decline (≥20%) in measured FEV1 value from the reference (baseline) value. The baseline value is computed as the mean of the best 2 post-operative FEV1 measurements (taken >3 weeks apart). CLAD can present either as a predominantly obstructive ventilatory pattern (BOS, bronchiolitis obliterans syndrome), a restrictive pattern (RAS, restrictive allograft syndrome), or a mixed obstructive and restrictive pattern.[9]

[1]	Snell GI, Yusen RD, Weill D, Strueber M, Garrity E, Reed A, et al. Report of the ISHLT Working Group on Primary Lung Graft Dysfunction, part I: Definition and grading-A 2016 Consensus Group statement of the International Society for Heart and Lung Transplantation. J Heart Lung Transplant 2017;36:1097–103. https://doi.org/10.1016/j.healun.2017.07.021.

[2]	Greenland JR, Perch M, Halloran K, Levine DJ, Morrell ED, Reed A, et al. Considerations for Endpoints in Lung Transplant Clinical Trials: An ISHLT Consensus Statement. J Heart Lung Transplant 2026;45:e104–28. https://doi.org/10.1016/j.healun.2025.09.017.

[3]	Messika J, Belousova N, Parquin F, Roux A. Antibody-Mediated Rejection in Lung Transplantation: Diagnosis and Therapeutic Armamentarium in a 21st Century Perspective. Transplant International 2024;37. https://doi.org/10.3389/ti.2024.12973.

[4]	Levine DJ, Glanville AR, Aboyoun C, Belperio J, Benden C, Berry GJ, et al. Antibody-mediated rejection of the lung: A consensus report of the International Society for Heart and Lung Transplantation. J Heart Lung Transplant 2016;35:397–406. https://doi.org/10.1016/j.healun.2016.01.1223.

[5]	Todd JL, Neely ML, Kopetskie H, Sever ML, Kirchner J, Frankel CW, et al. Risk Factors for Acute Rejection in the First Year after Lung Transplant. A Multicenter Study. Am J Respir Crit Care Med 2020;202:576–85. https://doi.org/10.1164/rccm.201910-1915OC.

[6]	Stewart S, Fishbein MC, Snell GI, Berry GJ, Boehler A, Burke MM, et al. Revision of the 1996 working formulation for the standardization of nomenclature in the diagnosis of lung rejection. J Heart Lung Transplant 2007;26:1229–42. https://doi.org/10.1016/j.healun.2007.10.017.

[7]	Liu J, Jackson K, Weinkauf J, Kapasi A, Hirji A, Meyer S, et al. Baseline lung allograft dysfunction is associated with impaired survival after double-lung transplantation. J Heart Lung Transplant 2018;37:895–902. https://doi.org/10.1016/j.healun.2018.02.014.

[8]	Li D, Weinkauf J, Kapasi A, Hirji A, Varughese R, Lien D, et al. Baseline lung allograft dysfunction in primary graft dysfunction survivors after lung transplantation. Respir Med 2021;188:106617. https://doi.org/10.1016/j.rmed.2021.106617.

[9]	Verleden GM, Glanville AR, Lease ED, Fisher AJ, Calabrese F, Corris PA, et al. Chronic lung allograft dysfunction: Definition, diagnostic criteria, and approaches to treatment-A consensus report from the Pulmonary Council of the ISHLT. J Heart Lung Transplant 2019;38:493–503. https://doi.org/10.1016/j.healun.2019.03.009.
